# New insights on the pathogenesis of endometriosis and novel non-surgical therapies

**DOI:** 10.4274/jtgga.2018.0090

**Published:** 2018-08-06

**Authors:** Anom Suardika, Tjokorda Gede Astawa Pemayun

**Affiliations:** 1 Department of Obstetrics and Gynecology, Udayan University, Sangah Hospital, Bali, Indonesia

**Keywords:** Endometriosis, novel therapy, non-invasive

## Abstract

Endometriosis is a disease of theories, but none has succeeded to explain the whole picture. Most widely available drugs for endometriosis aim to relieve symptoms and improve fertility. Unfortunately, many short and long-term side-effects are associated with the treatments. To overcome this problem, researchers have developed many novel therapeutic agents, including non-invasive technique. We aim to provide new insights on pathogenesis model and novel non-surgical treatments for endometriosis, including drugs already available in the market and also drugs which are still under research. Seven novel treatment modalities are recognized, namely dienogest, aromatase inhibitor (AI), gonadotrophine-releasing hormone (GnRH) antagonist, anti tumor necrosing factor (TNF)-α, selective estrogen receptor modulator (SERM), selective progesterone receptor modulator (SPRM), and high-intensity focused ultrasound (HIFU). Dienogest, AI, and GnRH antagonists are effective novel treatments with good tolerance and safety. SERM and SPRM show inconsistent results, while anti-TNF-α is still in the animal experimental stage. HIFU is a potential futuristic treatment. However, it is still a long way until this technology is truly applicable.

## Impacts of Practice

* Knowledge on new pathogenesis and pathophysiologic models of endometriosis may modify clinicians’ perspective on therapy

* Implementing new therapeutical options may help to improve patients’ satisfaction

## Introduction

Endometriosis is an estrogen-dependent chronic inflammatory disease associated with chronic pelvic pain and infertility. Endometriosis causes a wide spectrum of symptoms and inflicts heavy socio-economic burden to patients. Endometriosis occurs in about 2-10% of women of reproductive age (1,2) and approximately in 50% of infertile women (3). The economic burden was reported 69,4 billion dollars in United States every year (4,5).

Clinical diagnosis of endometriosis is often difficult due to the wide spectrum of symptoms which most are non-spesific. Visual observation through laparoscopy and hystopathological sampling are the gold-standards (2,6). The most common complaints in endometriosis patients are dysmenorrhea (79%) and chronic pelvic pain (69%) (1). Many theories have been proposed as the basis for medical treatment (7,8,9,10). Conventional medical treatments include progesterone, danazole, combined oral contraceptive (COC), gonadotrophine-releasing hormone (GnRH) agonist, and non-steroid anti-inflammatory drugs (NSAIDs). The aim of these conventional therapies are suppression of inflammatory reaction, reduction of serum estrogen level, or increasing serum progesterone level (1,9,10).

The efficacies of conventional therapies are good, but when given for a longer period, some aspects should be considered: 1) significant potential side-effects, especially for reproductive-aged women as the result of hipoestrogenic environment; 2) high relapse rate despite optimal medical therapy, and 3) costly treatments (9,10). Along with the massive development in the etiopathogenesis theories, many treatment modalities emerge, aiming at specific molecular mechanism and to avoid previous generations of drugs’side-effects (1,11). In this paper, we present novel therapies for endometriosis and their specific mechanisms of action.

## Overview of Endometriosis Pathogenesis

Among existing theories on endometriosis pathogenesis, Sampson’s retrograde menstruation theory is the most popular, because it is scientifically proven, easy to understand and widely acceptable. The theory is supported by laparoscopic findings from women on perimenstrual period, of which menstrual blood components were found in peritoneal cavity on 90% of patients (12,13,14,15). In 1960s, Ferguson proposed that mesothelial cells from peritoneal and ovarian surfaces may undergo metaplasia and transform into endometrial tissue (12,15,16,17). Consistently, mullerian remnant theory also describes that primordial cells spread accross posterior pelvic wall may transform into endometrial tissue when exposed to high-level estrogenic stimulus (12,16). Stem cell potential to differentiate into endometrial tissue under hyperesterogenic influence has also been studied (15,18).

In endometriosis, various biomolecular changes are involved in the development of lesions, including: impaired immune system response, increased cytokines and pro-inflammatory mediators, increased angiogenic activity, excessive estrogen production, and progesterone resistance. Ectopic tissues may avoid normal apoptotic and phagocytosis mechanisms, presumably due to decreased expression of metalloproteinases, CD36 and increased production of dissolved intercellular adhesion molecule-1 (19).             

Increased inflammatory activity is also present in endometriosis, through the overproduction of Interleukin (IL)-1, IL-6, IL-8, monocyte chemo-attractant protein-1, RANTES, tumor necrosis factor (TNF)-α and TNF-β. These mediators will further stimulate the prostaglandins production and triggers the release of vascular endothelial growth factor that serves as pro-angiogenic agent (19).

**Figure 1:**
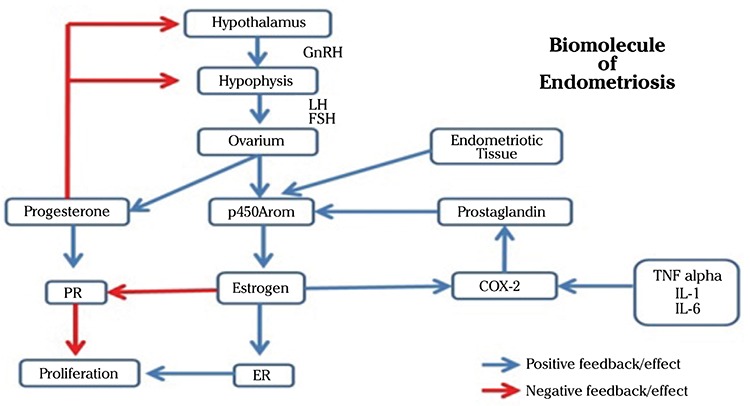
Biomolecular processes in endometriosis *PR: Progesterone receptor; ER: Estrogen receptors; IL: Interleukin; COX: Cyclooxygenase; LH: Luteinizing hormone; FSH: Follicle-stimulating hormone, GnRH: Gonadotrophine-releasing hormone; TNF: Tumor necrosing factor*

The most important factor in the pathophysiology of endometriosis is the estrogen hormonal dysregulation and progesterone resistance. Hypomethylation of the CpG cluster changes the balance of estrogen receptors, from alpha subtypes (ERα) dominance into beta subtypes (ERβ) dominance. In endometrial tissue, ERβ binds to the promoter of ERα, suppressing the production of ERα, thereby reducing the formation of progesterone (PR) receptor, resulting in resistance to progesterone. ERβ regulates cell cycle progression, and contributes to the proliferation of endometriotic cells (20,21). Prostaglandins are also known to increase the activity of steroidogenic proteins especially aromatase (p450arom) and the production of tissue estrogens, thereby aggravating the condition (22,23). We can see the summary of biomolecular process of endometriosis in Figure 1.

An understanding of the biomolecular processes in endometriosis has now brought about the possibility of potential new therapies. These new therapies aim specific pathophysiologic mechanisms that have not been targeted by conventional methods. Although promising, some has not been fully tested in humans, and some are still in the early phase of clinical trials (24,25). 

## Novel Medical Therapies

### Dienogest

Dienogest (DNG) is an oral progestin that has been recognized as single-drug therapy for endometriosis in Europe, Japan, Australia and Singapore (26,27). DNG is a 19-nortestosterone derivative with the advantage of short plasma half-life, strong progestin effect on endometrium, high bioavailability, anti-androgenic activity, and moderate gonadotropin secretion inhibition, with no interference with p450 cytochrome in the liver (28,29). Inhibition of gonadotropin secretion is not as high as GnRH agonist, with mean estrogen level maintained at 30-60 pg/mL (28).

DNG 2 mg/day has been shown to significantly inhibit the expression of genes and proteins associated with aromatase and cyclooxygenase (COX)-2, as well as prostaglandin E2 (PGE2) production (30,31). DNG administration also increases the PR-β/PRα ratio, as well as decreases the ERb/ERa ratio; thus, minimizing progesterone resistance in endometriosis patients (32). Provision of long-term DNG has been proven to be effective, safe, tolerable, as well as low incidence of adverse events and drop-out rates (26,33). DNG administration, when compared to GnRH agonists, provides a similar improvement in the intensity of complaints, but lower decrease in estrogen level or negative impact on bone mass (26). DNG can be tolerated in long-term administration due to negligible antiestrogenic, glucocorticoid, and mineralocorticoids effects (26,29). The most frequent side effects are breast pain (4.2%), nausea (3.0%), and irritability (2,4%) (27,34).

### Aromatase inhibitor

The administration of aromatase inhibitors (AI) in endometriosis patients may directly decrease aromatase activity in endometriotic tissue and estrogen level, thereby suppressing COX-2 activity, decreasing PGE2 level, and breaking the positive feedback loop (35,36,37,38). When given to premenopausal women, AI suppresses estrogen production and increases the follicle stimulating hormone (FSH) production by the pituitary gland; dosage of 0.5 mg decreases estrogen up to 97-99% (35). The third-generation AIs are selective, reversible, and potent triazole derivatives, making it suitable for use in clinical practice (35). The recommended daily dose is 1 mg for anastrozole, 2.5 mg for letrozole and 25 mg for exemestane, with the lowest decrease in E2 levels caused by exemestane (52-72%) (39).

AIs when combined with progestogen, COC, or GnRH agonist significantly decrease endometriotic pain intensity, thereby improving patient’s quality of life. AI is superior in preventing postoperative recurrence when compared to GnRH or Danazol, within 6 months period (40,41). AI is equivalent to clomiphene citrate in increasing pregnancy rates (42). In post-menopausal patients, AI shows exellent performance (43). Side effects are mostly mild (ie mild headache, joint pain or stiffness, nausea, diarrhea, hot flashes, mild bone density decrease) (40,41).

### GnRH antagonist

GnRH antagonists act by competitively block GnRH receptor. When compared to GnRH agonist, this class of drugs shows no-flare period, faster therapeutic onset, and unchanged pituitary sensitivity to GnRH after discontinuation of therapy (44,45,46,47,48). Single dose elagolix of 25-400 mg will decrease luteinizing hormone up to 22-35%, FSH 62-71%, and estradiol 42-65% (46). Administration of Elagolix 150 mg per day (75 mg twice daily) improves pelvic pain as measured with Biberoglu and Behrman pain scale, comparable to DMPA injection (47). The highest improvement on patient’s quality of life as measured by Endometriosis Health Profile-5 attained at dosage 150 mg per day (49,50).

The most common side effects are hot flush, nausea and headache. With long-term use up to 6 months, these side effects are increased by 10%. Approximately 25% of patients become amenorrhea after 8 weeks of therapy with a dose of 150 mg per day, but this number decreases to 7.6% after 24 weeks (44). Elagolix causes a mild decrease in axial bone density (44,47). The rate of pregnancy increases by 5% at a dose of 150 mg per day (47). No teratogenic effect was found from elagolix treatment (44).

### Anti-TNF-α

As noted earlier, TNF-α has a major role in the pathogenesis and survival of endometriosis lesions. Thus, targeting this molecule is a rational approach to treat endometriosis. Drugs classified as anti-TNF-α are either monoclonal antibodies (infliximab) or soluble TNF-α receptors (etanercept, TNF recombinant human protein bindings) (51,52,53). In baboons, anti-TNF-α inhibits the development of lesions significantly, but fails to increase pregnancy rates, fecundity levels per cycle, time to pregnancy, and cumulative pregnancy rates (54,55). *In vitro* studies have shown that regression of lesion size, as well as decreased expression of inflammatory cytokines after anti-TNF-α administration (56,57,58,59,60,61). Mild side-effects may include headache and allergic reactions during intravenous administration, whereas long-term administration is associated with serious infections and tuberculosis reactivation (51,62).

### Selective Estrogen Receptor Modulator

The selective estrogen receptor modulator (SERMs) are agents that have the effect of estrogen antagonists on the target organ, and the agonistic effects on bones and blood vessels (63,64). There are three types of SERM: triphenylethylene (tamoxifen), benzothiophene (raloxifen), and steroid (63). In animal models, raloxifene showed comparable benfits with anastrozole (AI) in reducing the size of lesion (65). In humans, the results are still unsatisfactory (64,66). Newer generation SERM, bazedoxifen (BZA), is being extensively studied for endometriosis therapy (47,64). The decrease in the size of lesions & reduced expression of various genes involved in tissue proliferation are significantly found after the administration of BZA 3 mg/kg/day (64,67). BZA administration alone (3 mg/kg/day) or BZA-conjugated-estrogen combination led to lesion size reduction and decreased ER expression (68).

### Selective Progesterone Receptor Modulator

Selective progesterone receptor modulator (SPRMs) are PR ligands with specific clinical effects: agonists, antagonist, or agonist-antagonist combination on progesterone target tissues *in vivo* (69). The ideal SPRM for therapy is capable of triggering antiproliferative effects on the endometrium and breast, but retains the protective effects of estrogen on bone and cardiovascular systems (69,70,71). Histologic observation shows that SPRM administration results in reduced endometrial thickness, loss of mitotic activity, and increased stromal density (71,72). In animals, SPRM does not produce ovarian estrogen production suppression. It seems like the suppressive effects are stronger on endometrial tissue compared to hypothalamus-pituitary-gonad axis (71).

Experimental study on primates by giving asoprisnil and asoprisnil ecamate, resulted in amenorrhoea, endometrial proliferative suppression, and endometrial atrophy (69). In phase II studies, asoprisnil of 5, 10 and 25 mg doses significantly improved the non-menstrual pelvic pain scores (69,73). In a study on rats, ulipristal administration reduced endometriotic focci by at least 50% and is associated with a decrease in the number of cells exhibiting proliferative activity (70,74,75). In humans, administration of ulipristal acetate (doses 10, 50 or 100 mg) in the mid-luteal phase inhibits endometrial maturation, decreases endometrial thickness, and induces endometrial atrophy. Also, endometrial glands shows mixed secretory and proliferative characteristics (76,77).

## Non-Invasive Therapy

### High-Intensity Focused Ultrasound

High-intensity focused ultrasound (HIFU) is a new technique that utilizes local heating phenomenon. This technique was first introduced by Zhang and Wang (78) in 1940 (79). Currently, HIFU can be performed with the guidance of ultrasound (USgHIFU) or magnetic resonance imaging (78). The physical basis of HIFU technique is by focusing the ultrasonic wave so that high intensity acoustic energy will be absorbed and then converted into heat at a designed focal point, resulting in thermal coagulation. Other mechanisms that may be involved are acoustic cavitation (interaction of sound waves with microscopic gas formation) and radiation forces (microflow of liquid around the bubbles) (80,81).

Abnormal tissue ablation with USgHIFU in the case of adenomyosis provides good safety and effectiveness as well as significant improvement of clinical symptoms (82). HIFU has also been proven effective for ablation of endometriotic lesions. In one study, cyclic pain disappeared in all patients after 3-31 months (mean 18.7 months) (83). Some of the HIFU weaknesses are as follow: 1) ultrasonic waves can not penetrate hollow viscera, 2) time-consuming in certain cases, 3) movement during procedure is not allowed, thus, it needs additional regional anesthesia, which is the policy in many centers (79). Severe complications ever reported are post-procedure vaginal bleeding, and unexplained tumor enlargement that causes discomfort (84).

Endometriosis is a gynecologic disorder highly associated with chronic pelvic pain and infertility. Dienogest, AI, and GnRH antagonists have been proven effective as endometriosis therapy in many clnical studies, with good tolerance and safety. Studies on SERM and SPRM are mostly still in phase I and II clinical trials, that show inconsistent results. Anti-TNF-a is still studied in the animal model. HIFU is a potential futuristic treatment. However, it is still a long way until this technology is truly applicable.
